# Does Resistance Training Improve the Quality of Life of People With Parkinson's Disease? Evidence and Recommendations for Clinical Application Through a Systematic Review and Meta‐Analysis of Randomized Clinical Trials

**DOI:** 10.1002/pri.70266

**Published:** 2026-06-29

**Authors:** Loiane Cristina de Souza, Luiz Senna, Anderson D'Oliveira, Rubia Truppel, Guilherme Torres Vilarino, Alexandro Andrade

**Affiliations:** ^1^ Department of Physical Education Santa Catarina State University—UDESC Florianópolis Brazil; ^2^ Department of Physiotherapy Santa Catarina State University—UDESC Florianópolis Brazil

**Keywords:** modalities of physical exercise, Parkinson's disease, quality of life, resistance training

## Abstract

**Background:**

Parkinson's disease is a neurological condition with motor and non‐motor symptoms that negatively affect well‐being and quality of life.

**Objective:**

This review aimed to analyze the effects of resistance training on quality of life in individuals with Parkinson's disease and to summarize recommendations for clinical application.

**Data Sources:**

Following PRISMA recommendations, searches were conducted in Web of Science, PubMed, EMBASE (with ClinicalTrials.gov), PEDro, CINAHL, and Scopus.

**Study Selection:**

RCTs were included that compared the effects of resistance training with other therapeutic modalities or a control group on the quality of life in people with Parkinson's.

**Data Extraction and Synthesis:**

Data were extracted using Microsoft Excel and analyzed in RevMan. The risk of bias was assessed using the RoB 2 tool, the protocol reporting completeness was evaluated using CERT and certainty of the evidence was rated using GRADE.

**Results:**

Fourteen studies were included, featuring highly heterogeneous protocols. Resistance training was superior to control (SMD −0.81; CI −1.45 to −0.18; *I* ^2^ 88%; moderate certainty of the evidence), although no significant difference was found compared to other therapeutic modalities (SMD −0.02; CI −0.44 to 0.41; low certainty of the evidence). Exploratory subgroup analyses suggested a trend toward more favorable results in trials lasting longer than 12 weeks, involving patients with mild‐to‐moderate disease severity (H&Y ≤ 3), and reporting high protocol completeness (CERT > 11). Most of the studies were classified as having a low risk of bias and presented a moderate description of the protocols in the CERT.

**Limitations:**

The included studies exhibited high heterogeneity and required data imputation for missing standard deviations. Additionally, exploratory subgroup analyses were underpowered due to the limited number of trials.

**Conclusions:**

Current evidence suggests that resistance training may improve quality of life in individuals with Parkinson's disease. Exploratory subgroup analyses tentatively suggest potential trends favoring mild‐to‐moderate stages and intervention exceeding 12 weeks, while being a safe practice. However, high heterogeneity limits the certainty of the evidence. Future studies should prioritize standardized reporting (CERT) to ensure intervention reproducibility.

**Trial Registration:**

PROSPERO record (CRD42024588780)

## 1 Introduction

Parkinson's disease (PD) is a progressive neurodegenerative disorder characterized by the loss of dopaminergic neurons in the substantia nigra of the midbrain (Barbosa and Sallem 2005; Goetz 2011; Latif et al. 2021). Generally, the clinical diagnosis of PD is based on motor symptoms, including resting tremors, muscle rigidity, bradykinesia, and postural instability (Simon et al. 2020). In addition, non‐motor symptoms, such as pain, sleep disorders, anxiety, depression, cognitive decline, fatigue, and several other symptoms may emerge as PD progresses (Poewe and Mahlknecht 2009).

The accumulation of these symptoms substantially impairs perception of well‐being and quality of life (QoL) (Martinez‐Martin et al. 2011). QoL has been investigated as an important outcome in the clinical environment (Sieczkowska et al. 2019, 2020), and is considered the self‐perception of the physical, psychological, and social effects of the disease on their daily life, and therefore, it is a multidimensional construct requiring subjective and individual assessment (Martínez‐Martín 1998).

According to Ray et al. (2018), approximately 6.1 million people were living with PD in 2016. Furthermore, the prevalence of PD is expected to continue rising in the coming decades, demonstrating a growing need for effective treatments to promote QoL and well‐being in this population (Brienesse and Emerson 2013).

Currently, the most common treatments for PD involve the use of controlled drugs, such as levodopa, primarily aimed at managing motor symptoms and physical manifestations (Santos García et al. 2023). However, some motor symptoms, such as gait disturbances and bradykinesia, may not improve with medication alone, requiring approaches based on physical exercise (Brienesse and Emerson 2013; Santos García et al. 2023).

The current literature points to physical exercise, across a wide range of modalities, as a beneficial intervention for the physical and psychological health of older adults (Di Lorito et al. 2021; da Cruz et al. 2022; D’Oliveira et al. 2022). Regarding PD, the evidence mainly highlights benefits related to physical symptoms, with physical exercise demonstrating effectiveness for strength gains, balance and gait improvement, cardiorespiratory fitness, and functional capacity (Brienesse and Emerson 2013; Ernst et al. 2023; Goodwin et al. 2008; Ramazzina et al. 2017). However, evidence remains limited regarding the effects of physical exercise on psychological symptoms and QoL, which is one of the main markers of the impact of the disease (Martinez‐Martin et al. 2011). A recent systematic review with network meta‐analysis by Ernst et al. (2023), aimed to compare the effects of different types of physical exercise on the health outcomes in people with PD, including QoL. The authors included 156 studies, of which 55 focused only on QoL, and found that physical exercise provided benefits to this outcome, however, when analyzing the RT modality, the data were inconclusive, highlighting the need for a more detailed analysis.

Another recent network meta‐analysis, published in 2025, found Qigong to be superior to other modalities for QoL, but did not provide detailed recommendations regarding the specific protocols of each modality (Y. Li et al. 2025). Thus, although RT presents positive results across different populations (Andrade et al. 2017; Albuquerque et al. 2022; Andrade et al. 2019), its specific application in PD remains insufficiently characterized. While previous reviews have examined exercise broadly or focused on outcomes other than QoL, few have conducted detailed subgroup analyses exploring how specific RT protocol characteristics may influence outcomes. Specifically, further exploration is needed to examine outcomes stratified by disease severity and to evaluate the completeness of RT protocol reporting using standardized tools, both of which are important to understand the contexts in which RT is applied and for assessing the reproducibility of reported interventions. This limitation restricts the translation of findings into clinical practice. A systematic review with detailed analyses targeting RT protocols, disease severity, and reporting completeness in relation to QoL in PD is therefore needed to provide more precise observations and to support informed clinical decision‐making.

Given the limited evidence regarding the effects of RT and its prescription characteristics in patients with PD, the current review aims to analyze the effects of RT on QoL in people with PD and summarize recommendations for clinical applications.

## 2 Method

### 2.1 Delimitation

This review follows the recommendations of the Preferred Reporting Items for Systematic Reviews and Meta‐Analyses (PRISMA) (Page et al. 2021) and the protocol was registered on the PROSPERO platform (CRD42024588780).

### 2.2 Search Strategies

Searches were conducted in November 2025 across six databases: Web of Science (main collection), PubMed, EMBASE (including clinicaltrials.gov), SCOPUS, CINAHL and PEDro without applying time restrictions. One researcher performed the search independently (LFS) and subsequently cross‐checked by a second researcher (LCS). Both researchers obtained identical results, confirming the reliability of the search strategy and ensuring that no relevant studies were overlooked at this stage.

To determine the descriptors, Mesh (Medical Subject Headings) and descriptors used in previous studies were adopted. Population terms (“Parkinson disease” OR Parkinsonian OR Parkinson OR “parkinson's” OR parkinsons) AND intervention (“Resistance Training” OR “Strength Training” OR “Resistance Exercise” OR “Muscle Strengthening”) were combined. The complete strategy used in each database can be found in Supporting Information S1: Section (A).

After searching the databases, a manual search was performed of references of the selected studies and in Google Scholar.

### 2.3 Eligibility Criteria

Eligibility criteria were defined using the PICOS method. Studies were included if participants had a diagnosis of PD; the intervention included RT as the main part of the intervention (it could also include mobility and balance exercises); the intervention group was compared with a control group, other exercises modalities, or other therapeutic modalities; QoL was assessed as an outcome; and the study was a randomized clinical trial. Studies were excluded if they included participants with other neurological diseases in the sample, the intervention included aerobic exercise or only power exercises, or they did not present comparisons with other groups. The eligibility process was carried out by two independent researchers; the first stage involved reading the titles and abstracts, followed by the full‐text reading stage. In cases of disagreement between the two reviewers, a third author was consulted (AD). This process was carried out using the Rayyan website. To determine the consistency between the screening of independent researchers, the Kappa coefficient was used, and the analysis was performed using SPPS version 20.0.

### 2.4 Data Extraction and Analysis

All data were extracted and verified by two authors (LCS and LFS) into a Microsoft Excel spreadsheet (2016—Microsoft Corporation, Redmond, USA). Information was collected on bibliometric data (author and year of publication), population characteristics (total number of participants, groups, dropouts, age range and mean, gender), RT protocol (professional who conducted the intervention, total duration, weekly frequency, intensity, load, number of repetitions, number of sets, time per session, and reported adverse events), outcome (instrument used, descriptive result, mean and standard deviation values pre and post‐intervention for the QoL outcome), and study (design and comparison group). The extraction was performed by one researcher and verified by another. These data are summarized in text and tables. The complete extraction of individual results from each study can be found in Supporting Information S1: Section (B).

Quantitative analysis was performed through a meta‐analysis, which followed the recommendations of the *Cochrane Handbook for Systematic Reviews of Interventions* (J. P. T. Higgins et al. 2024). Changes from baseline to post‐intervention (delta) and the SD were extracted from each study to perform the meta‐analysis, because some studies did not report SD for change scores, these were imputed using a correlation coefficient in accordance with Section 6.5.2.8 of the Cochrane Handbook. To ensure the robustness of the findings, a sensitivity analysis was conducted using an alternative correlation value. Calculations were performed using a random‐effects model and data are presented as standardized mean difference (SMD). To assess statistical heterogeneity of the treatment effect across studies, Cochran's Q test was used, with a *p*‐value of 0.1 considered statistically significant, and the *I* ^2^ inconsistency test, with values greater than 50% considered indicative of high heterogeneity. Effect size was according to Cohen's *d* (*d* = 0.2 was considered a small effect size, 0.5 a moderate effect size, and 0.8 a large effect size) (J. P. T. Higgins et al. 2024). The results of the intervention group were compared with the results of the control group or another group of other therapeutic modalities (when applicable), including subgroup analyses: study duration (planned a priori), disease staging by H&Y, and protocol reporting by CERT, both conducted on an exploratory basis. Additional priori subgroup analyses were planned but could not be performed due to insufficient data, including analyses by session duration, weekly training frequency, and load progression in RT. Meta‐analyses were performed in RevMan, version 5.4. The complete extraction of mean and standard deviation values of QoL before and after RT intervention in patients with Parkinson's disease can be found in Supporting Information S1: Section (C).

### 2.5 Risk of Bias (ROB2), Consensus on Exercise Reporting Template (CERT) and Certainty of Evidence

The Cochrane Risk of Bias for Randomized Controlled Trials (Rob2) tool was used to analyze the risk of bias of the studies (J. P. Higgins et al. 2019). The Rob2 measures five domains that may present systematic errors in the performance of studies and compromise the reliability of the results, namely: bias resulting from the randomization process (D1), bias due to deviations from the intended interventions (D2), bias due to missing outcome data (D3), bias in the measurement of the outcome (D4), and bias in the selection of the reported result (D5). A Microsoft Excel spreadsheet (2016—Microsoft Corporation, Redmond, USA) standardized for RoB2 was used for the analysis. Two researchers performed the analysis independently (LCS and LFS), which was checked by two other researchers (AD and GTV).

The CERT tool was used to assess the standardization of reporting of interventions with physical exercise, considering minimum criteria for replication. This tool consists of 16 items (19 topics) that do not imply the methodological quality of the studies but make it possible to verify how replicable the intervention is (Slade et al. 2016). Each topic that is reported adequately receives a score of “1” and when inadequately reported it receives a score of “0”, so the final score can vary from 0 to 19. One researcher performed the analysis (LFS) and a second checked the score (LCS).

The certainty of evidence was determined using Grading of Recommendations Assessment, Development, and Evaluation (GRADE) (Neumann and Schünemann 2024) and GRADEproGDT (https://gdt.gradepro.org).

## 3 Results

### 3.1 Selection of Studies

**FIGURE 1:**
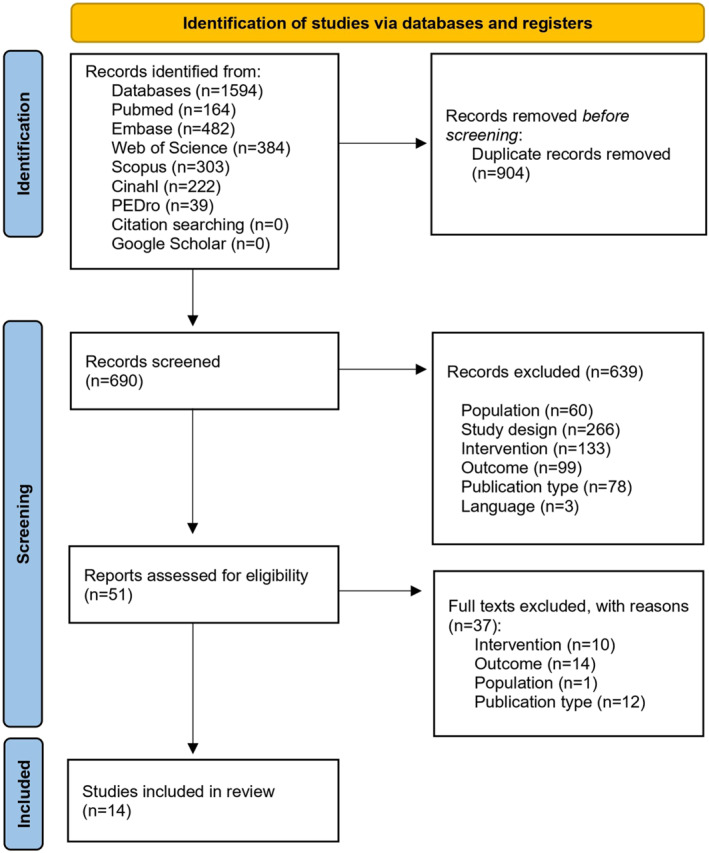
PRISMA flowchart of study identification.

The initial search across six databases resulted in 1594 records. After the removal of duplicate records, 690 articles remained for screening. In the first screening stage, 639 studies were excluded based on title and abstract screening. Following full‐text review of the remaining 51 articles, 14 studies were included. During the independent screening phase, a kappa coefficient of 0.615 was obtained, indicating moderate‐to‐substantial agreement. Any disagreements between the two primary reviewers were resolved through consultation with a third reviewer. The details of the study selection are presented in Figure 1.

### 3.2 Characteristics of Selected Studies

Among the included studies, the oldest was published in 2014 (F. Li et al. 2014) and the most recent in 2021 (J. Chen et al. 2021; Strand et al. 2021). Of these, five were carried out in Brazil (J. Chen et al. 2021; de Lima et al. 2019; Ferreira et al. 2018; Silva‐Batista et al. 2016; Smaili et al. 2018), three in the United States of America (F. Li et al. 2014; Strand et al. 2021; Cherup et al. 2019), two in Australia (Morris et al. 2015, 2017), and one each in China (Kwok et al. 2019), Belgium (Demonceau et al. 2017), Spain (Santos et al. 2017), and Germany (Schlenstedt et al. 2015). A total of 1088 men and women diagnosed with PD were randomized across the included studies. The mean age ranged from 63.2 ± 6.4 years (J. Chen et al. 2021) to 75.7 ± 7.2 years (F. Li et al. 2014).

The exercise modalities used as comparators to RT included multimodal training (strength, power, and functional) (Strand et al. 2021), power training (Cherup et al. 2019), mindfulness yoga (Kwok et al. 2019), neurofunctional training (Smaili et al. 2018), aerobic training (Demonceau et al. 2017), RT with instability (Silva‐Batista et al. 2016), movement strategy training (Morris et al. 2015), balance training (Schlenstedt et al. 2015), Tai Chi (F. Li et al. 2014), and stretching (F. Li et al. 2014; J. Chen et al. 2021). Control groups were also used as comparators, two involved life skills interventions (Morris et al. 2015, 2017), while the remaining groups received no intervention (Silva‐Batista et al. 2016; Demonceau et al. 2017; Santos et al. 2017).

The studies included assessed QoL using the PDQ‐39 or PDQ‐8. Only three trials reported all eight PDQ‐39 domains (J. Chen et al. 2021; Smaili et al. 2018; Demonceau et al. 2017); the others reported total scores only.

### 3.3 Characteristics of Resistance Training Interventions

The RT intervention parameters exhibited high heterogeneity, with considerable variation in duration, frequency, and prescribed intensity. Regarding training frequency, most studies prescribed sessions twice per week (F. Li et al. 2014; J. Chen et al. 2021; de Lima et al. 2019; Ferreira et al. 2018; Silva‐Batista et al. 2016; Smaili et al. 2018; Cherup et al. 2019; Morris et al. 2015; Santos et al. 2017; Schlenstedt et al. 2015), three studies prescribed once‐weekly sessions (Morris et al. 2015, 2017; Kwok et al. 2019), and one prescribed three times per week (Strand et al. 2021). One protocol (Demonceau et al. 2017) alternated between two and three weekly sessions. Intervention duration ranged from 6 weeks (Morris et al. 2017) to 24 weeks (F. Li et al. 2014).

The exercise prescriptions across interventions aimed to strengthen the major muscle groups of the upper and lower limbs, including the pectoralis major, latissimus dorsi, quadriceps, hamstrings, plantar flexors, gluteal, and abdominal muscles. Exercises were performed using machines (J. Chen et al. 2021; Strand et al. 2021; Cherup et al. 2019; Santos et al. 2017) or free weight such as dumbbells, barbells, ankle weights, weight vests, resistance bands or body weight (F. Li et al. 2014; J. Chen et al. 2021; de Lima et al. 2019; Ferreira et al. 2018; Smaili et al. 2018; Morris et al. 2015, 2017; Kwok et al. 2019; Schlenstedt et al. 2015). Two studies utilized a combined approach, incorporating both machines and free weights exercises in the same session (Silva‐Batista et al. 2016; Demonceau et al. 2017). Some interventions included only exercises targeting the lower limb and abdominal muscles (F. Li et al. 2014; Morris et al. 2015, 2017; Kwok et al. 2019; Schlenstedt et al. 2015).

Although most studies reported training volume, several did not clearly quantify training intensity (F. Li et al. 2014; Morris et al. 2015, 2017; Kwok et al. 2019; Schlenstedt et al. 2015). Rest intervals between sets were not reported in four studies (J. P. T. Higgins et al. 2024; Smaili et al. 2018; Morris et al. 2015; Demonceau et al. 2017), the remaining studies reported rest intervals ranging from 1 to 2 min. Only two protocols applied linear periodization, with progressive increases in intensity from 40% to 50% of one‐repetition maximum (1‐RM) to 80%–90% 1‐RM, accompanied by corresponding reductions in volume (Demonceau et al. 2017; Santos et al. 2017). One study adopted a similar linear progression based on target repetitions, prescribing intensity according to maximal or submaximal effort, progressing from 10–12 to 6–8 repetitions per set (Silva‐Batista et al. 2016). In the study of Smaili et al. (2018), the training load started at 1 kg dumbbells and progressed to 2 kg over the course of the sessions, whereas other studies maintained a constant intensity throughout the protocol, with loads between 50% and 80% 1‐RM (J. Chen et al. 2021; Strand et al. 2021; de Lima et al. 2019; Ferreira et al. 2018; Cherup et al. 2019). Across most protocols, training volume was relatively consistent, typically consisting of 2–3 sets of 6–15 repetitions.

Session duration typically ranged from 50 to 70 min, while two studies had shorter sessions of 30–40 min (de Lima et al. 2019; Ferreira et al. 2018), and one lasted 120 min (Morris et al. 2015). Most studies detailed the session components, with only four failing to report this information (de Lima et al. 2019; Ferreira et al. 2018; Smaili et al. 2018; Morris et al. 2015). Protocols typically comprised 5–10 min of warm‐up, 40–60 min of the main training component, and 5 min of relaxation. Sessions were held in community rehabilitation centers (F. Li et al. 2014; Morris et al. 2015; Kwok et al. 2019), and university facilities, including tracks and gyms (J. Chen et al. 2021; Strand et al. 2021; de Lima et al. 2019; Ferreira et al. 2018; Silva‐Batista et al. 2016; Smaili et al. 2018; Cherup et al. 2019; Demonceau et al. 2017; Santos et al. 2017; Schlenstedt et al. 2015). Only one study implemented a home‐based protocol (Morris et al. 2017).

Supervision was provided by physiotherapists (J. Chen et al. 2021; Silva‐Batista et al. 2016; Smaili et al. 2018; Morris et al. 2015, 2017; Demonceau et al. 2017) and physical education professionals (F. Li et al. 2014; de Lima et al. 2019; Ferreira et al. 2018; Santos et al. 2017). Some studies did not report the supervisors' professional qualifications, stating only that the intervention was delivered by a trained instructor or researcher (Strand et al. 2021; Cherup et al. 2019; Kwok et al. 2019). The characteristics of the interventions in each study are presented in Table 1.

**TABLE 1:** Characteristics of the interventions in each study.

Reference	Mean age by group (SD)	Intervention of each group	Duration (weeks)/frequency/time	Session components	Equipment/where it was performed	Muscle groups or exercises	Sets and repetitions	Intensity
Strand et al. (2021)	SPHG: 70.19 (9.06) SPG + Func: 68.63 (10.54)	SPHG: Strength, power and hypertrophy training SPG + Func: Strength training, power combined with functional circuits	12 weeks/3x/60 min	All sessions: Warm‐up + training + relaxation D1: Strength D2: Power D3: Hypertrophy (SPHG) versus Functional (SPG + Func)	SPHG: Keiser pneumatic machines. SPG + Func: Keiser pneumatic machines, medicine ball, agility ladder and cones.	Both groups: Leg/bench press, push‐up, seated row, hip abduction, puller, triceps/biceps pulley. SPG + Func adds: mobility, balance, and strength circuit	Both groups: D1: 3 × 8 reps D2: 3 × 6 reps D3: 3 × 12 reps (SPHG) versus 2‐3 × 4‐10 reps (SPG + Func)	Both groups: D1: 80% 1‐RM (90s rest) D2: 50% 1‐RM (120s rest) D3: 70% 1‐RM/60s rest (SPHG) versus Not monitored (SPG + Func)
J. Chen et al. (2021)	RTMG: 63.4 (6.9) RTFWG: 63.2 (6.4) CG: 63.6 (7)	RTMG: Resistance training with machines RTFWG: Resistance training with free weight CG: Stretching at home	12 weeks/2x/50 min	Warm‐up: 5 min—mobility exercises; training; stretches	RTMG: Gym/machinery RTFWG: Gym/free weight, bodyweight and elastic CG: At home	RTMG: Pulldown, trunk extension, seated row, seated bench press, supra sit‐up, and machine leg press RTFWG: Balance exercises, trunk stability, strengthening of the abdominal, paraspinal, middle trapezius, latissimus dorsi, rhomboid, quadriceps femoris and glutes muscles. CG: Stretches involving the trunk, calves, pectorals, triceps brachii and quadriceps.	RTMG/RTFWG: 1–3 sets, 8–12 reps, CG: 1 set per 15 s	RTMG/RTFWG: 60%–80% (1‐RM), 1–3 min rest between sets
Cherup et al. (2019)	STG: 69.3 (10.5) PTG: 73 (6.8)	STG: Strength training PTG: Power training	12 weeks/2x/60 min	STG/PTG: Warm‐up; strength/power training; relaxation.	STG/PTG: Keiser pneumatic machines	STG/PTG: Leg press, seated row, plantar flexion, seated pull, hip abduction, biceps curl, triceps extension, press, and seated bench press.	STG/PTG: 3 sets of 10 reps	STG: 70% (1‐RM), 1.5–2 min rest between sets PTG: 50% (1‐RM), 1.5–2 min rest between sets
Kwok et al. (2019)	YMG: 63.7 (8.2) RTSG: 63.5 (9.3)	YMG: Mindfulness yoga program for people with PD RTSG: Resistance training combined with stretching	8 weeks/1x/60–90 min	YMG: Yoga training (60 min); breath control (15 min); mindfulness meditation (15 min) RTSG: Warm‐up (10 min): Sitting and standing mobility exercises; resistance training; relaxation: Stretches (5 min) of the lower limbs.	YMG/RTSG: Rehabilitation center/group classes	YMG: 12 sun salutation poses and their variations RTSG: Weeks (1–2): Seated knee extension, seated knee flexion, hip flexion, alternating hip flexion, partial squat (30°) Weeks (3–4): Seated hip flexion with knee extended, lower limb circumference seated, plantar flexion standing, partial squat (45°). Weeks (5–6): Seated lower limb circumference, alternating cross reach, single‐leg balance, unilateral plantar flexion, isometric squat. Weeks (7–8): One‐leg balance, unilateral plantar flexion, lateral squat, and isometric wall squat	YMG: Not informed in the article RTSG: 2 sets of 20–30s per exercise, with 10s of rest between exercises	YMG/RTSG: Not reported
de Lima et al. (2019)	RTG: 66.2 (5.5) CG: 67.2 (5.2)	RTG: Resistance training CG: Pharmacological intervention only	20 weeks/2x/30–40 min	Resistance training (does not inform if there was a warm‐up and/or relaxation)	University resistance training lab/free weights and bars	Bench press, deadlift, unilateral row, standing plantar flexion and infra‐abdominal	2 sets of 8–12 reps	Submaximal/maximal exertion between 8 and 12‐RM, with 1–2 min rest between sets
Ferreira et al. (2018)	RTG: 64.1 (7.0) CG: 67.6 (8.9)	RTG: Resistance training CG: Pharmacological intervention only	20 weeks/2x/30–40 min	Resistance training (does not inform if there was a warm‐up and/or relaxation)	University resistance training lab/free weights and bars	Bench press, deadlift, unilateral row, standing plantar flexion and infra‐abdominal	2 sets of 8–12 reps	Submaximal/maximal exertion between 8 and 12‐RM, with 1–2 min rest between sets
Smaili et al. (2018)	RTG: 67.0 (7.9) NTG: 68.5 (6.5)	RTG: Resistance muscle training NTG: Neurofunctional training	12 weeks/2x/60 min	RTG: Resistance training (does not inform if there was a warm‐up and/or relaxation) NTG: Neurofunctional training (does not inform if there was warm‐up and/or relaxation)	RTG: Training room/dumbbell use NTG: Training room/balance pad, step, trampoline and swiss ball.	RTG: Strengthening and stretching exercises of the main muscles of the lower limbs and trunk. The exercises were performed standing, lateral decubitus, supine and sitting^a^ NTG: Exercises focused on gains in agility, balance, motor coordination between lower and upper limbs and trunk, as well as walking circuits^a^	RTG: 2 sets of 10 reps NTG: Not reported	RTG: Session 1–8: Weight 1 kg; Session 9–16: Weight 1.5 kg; Session 17–24: Weight of 2 kg. NTG: Progression was performed every 7 sessions, increasing the difficulty and quantity of the exercises
Demonceau et al. (2017)	ATG: 65 (8) STG: 67 (10) CG: 63.3 (6)	ATG: Aerobic training STG: Strength training CG: Standard care, no intervention	12 weeks/2‐3x/60–90 min	Warm‐up: 5–10 min—dynamic exercises; Training: Aerobic or strength; Relaxation: Stretches	ATG: Indoor stationary bike STG: Gym/machines and dumbbells	ATG: Training on the stationary bike STG: Extension chair, flexor chair, puller, plantar push‐up on machine, leg press, press, biceps curl and sit‐ups	ATG: Week 1–30 min 2‐4–30 to 40 min Week 6–12 – HIIT (30–180s/30–90s) or 40–45 min STG: Week 1–5: 2–3 sets, 10–15 reps Week 6–12: 2–3 sets, 5–8 reps	ATG: Week 1%–50% (MWL) Week 2%–4%–50%–55% (CMT) Without 6–12 – HIIT (70%–80% CMT/50% (MWL) or 40%–45% (MWL) STG: Week 1–5: 50%–60% (1‐RM) Week 6–12: 80%–90% (1‐RM)
Morris et al. (2017)	RTG: 71 (9) CG: 71 (10)	RTG: Progressive resistance training CG: Life skill program	6 weeks/1x/60 min (+60 min unsupervised)	RTG: Progressive resistance training; movement strategy training; Education in falls. The strength‐training component was prioritised	Weights, vest with weights and elastic bands/patient's house	RTG: Climbing the step, plantar flexion, sitting and standing, standing hip abduction, trunk rotation and extension. *Movement strategy:* Lifting and lying on the floor, walking, lifting and reaching, walking while holding objects, turning and mobility in bed	RTG: 2 sets, 8–12 reps, 2 min rest between sets. *Movement Strategy:* Not reported	Not reported
Santos et al. (2017)	RTG: 73.38 (8.81) CG: 73.80 (7.05)	RTG: Progressive resistance training CG: No intervention	8 week/2x/60–70 min	Warm‐up: 5–10 min—dynamic exercises and passive stretching; Training: 30–35 min—progressive resistance; Relaxation: 5–10 min—passive stretches	Gym/machines	Extension chair, flexor chair, machine bench press, front puller, back puller and seated row.	Week 1:2 circuits, 15–20 repetitions; 8–10 min rest. Week 3–6: 2 sets, 7–10 repetitions; rest 90–120s. Week 7–8: 2 sets, 4–7 repetitions. Rest 100–140s between sets	Week 1–6: 40%–50% (1‐RM); Week 7–8: 80%–85% (1‐RM)
Silva‐Batista et al. (2016)	RTG: 64.1 (9.1) RTIG: 64.2 (10.6) CG: 64.2 (8.3)	RTG: Resistance training RTIG: Resistance training with instability CG: No intervention	12 weeks/2x/50 min	Warm‐up: 10 min—stationary cycle bike (20–40 rpm) Training: Resistance training or resistance training with instability	Gym/machines, dumbbells, Swiss balls, Bosu, and balance discs	Leg press, front puller, plantar flexion, bench press on the machine and partial squat.	Month 1: 2‐3 sets of 10–12 repetitions, with 2 min rest; Month 2: 3–4 sets of 8–10 repetitions, with 2 min rest; Month 3: 4 sets of 6–8 repetitions, with 2 min of rest;	Month 1: Submaximal/maximal exertion between 10‐12‐RM Month 2: Submaximal/maximal effort between 8‐10‐RM Month 3: Submaximal/maximal effort between 6‐8‐RM
Morris et al. (2015)	RTG: 67.4 (10.4) MSTG: 68.4 (9.9) CG: 67.9 (8.4)	RTG: Progressive resistance training MSTG: Movement strategy training CG: Life skills	8 weeks/1x/120 min	Does not report whether there was warming or relaxation	Room in rehabilitation center/steps, vest with weights and elastic bands	RTG: Sit‐to‐stand exercises, trunk rotation, lateral pelvic control, step climbs, plantar flexion, tibialis anterior on the wall and sit‐ups. MSTG: Tasks involving walking, turning, reaching and sitting, sitting and standing, transferring from one chair to another, getting out of bed, and walking with multitasking	RTG: 1–2 sets of 8–15 repetitions. It does not report the rest time. MSTG: Not reported	RTG: Progression of repetitions, increase in sets, increase in overload in relation to body weight, or increase in task difficulty if the patient reports a subjective perception of effort less than 5. MSTG: Not reported
Schlenstedt et al. (2015)	RTG: 75.7 (5.5) BG: 75.7 (7.2)	RTG: Resistance training BG: Balance training	7 weeks/2x/60 min	Warm‐up: 10 min Main training: 50 min	RTG: Shin guards and elastic bands. BG: Balance cushion, balance disc and prospective balance sheet.	RTG: Squat, shin guard knee extension, plantar flexion, seated elastic hip flexion, seated shin guard hip flexion, standing hip abduction, and hip extension. BG: Week 1–4: Tandem positional balance, single‐leg balance, bipedal swing frontal, lateral and posterior tilt and balance with external disturbance. Week 4–7: Bi‐ and unipedal balance on unstable surfaces and gait training with obstacles.	RTG: 3 sets of 15–20 repetitions, with a two‐minute break between sets. BG: 3 sets, 45 s, with a 2‐min break between sets	RTG: Increased overload if you were able to perform more than 20 repetitions. BG: Week 1–4–normal surface Week 4–7–unstable surface.
F. Li et al. (2014)	RTG: 68 (8) TCG: 69 (9) SG: 69 (9)	RTG: Resistance training TCG: Tai Chi training SG: Stretching workout	24 weeks/2x/60 min	Warm‐up: 5–10 min; Training: Resistance, Tai Chi or stretching; Relaxation: 5 min	RTG: University setting/community center. Free weights and ankle weights. TCG: University setting/community center. Does not use equipment. SG: University set/community center. Chairs.	RTG: Lateral and frontal lunge, squat, lateral and frontal lunge, dorsiflexion and plantar flexion. TCG: Protocol with 6 Tai Chi movements integrated into an 8‐way routine. SG: Sitting and standing stretches involving upper limbs (neck, trapezius, shoulders, pectorals, and arms) and lower limbs (quadriceps, hamstrings/calves, and glutes)	RTG: 1–3 sets of 10–15 repetitions, do not inform if there was a break between sets. TCG: Not reported SG: Not reported	RTG: No external load until week 10, then gradually increase 1%–2% of body weight every 5 weeks up to 5% of body weight. TCG: Not reported SG: Not reported

Abbreviations: 1‐RM, one‐repetition maximum; ATG, aerobic training group; BG, balance training group; CG, control group; MSTG, movement strategy training group; MWL, maximum working load (W); NTG, neurofunctional training group; PTG, power training group; RT, resistance training; RTFWG, resistance training with free‐weight group; RTG, resistance training group; RTIG, resistance training with instability group; RTMG, resistance training with machine group; RTSG, resistance training and stretching group; SG, stretching group; SPG + Func, strength and power group combined with functional training; SPHG, strength, power, and hypertrophy group; STG, strength training group; TCG, Tai Chi group; YMG, yoga mindfulness group.

^a^ Short description of the intervention provided in the original study.

Regarding safety, few adverse events were reported, suggesting that RT is a safe modality, consistent with the comparator interventions included. The most common adverse event reported was joint pain, which was predominantly transient. Although adherence data were underreported, available findings indicated moderate‐to‐high compliance, generally accompanied by low attrition rates.

### 3.4 Effects of Resistance Training on the Quality of Life of People With PD

Most studies reported significant improvements in the QoL in people with PD following the RT intervention (F. Li et al. 2014; J. Chen et al. 2021; de Lima et al. 2019; Ferreira et al. 2018; Smaili et al. 2018; Cherup et al. 2019; Demonceau et al. 2017; Santos et al. 2017), while six trials found no significant pre‐ to post‐intervention differences (Strand et al. 2021; Silva‐Batista et al. 2016; Morris et al. 2015, 2017; Kwok et al. 2019; Schlenstedt et al. 2015). Among studies reporting all PDQ‐39 domains, the most consistent improvements were observed in mobility and activities of daily living (J. Chen et al. 2021; Smaili et al. 2018). A description of individual study results is provided in Supporting Information S1: Section (B). Regarding clinical relevance, the Minimal Clinically Important Difference (MCID), is defined as a reduction of 4.72 in the PDQ‐39 total score and 5.94 in the PDQ‐8 total score (Horváth et al. 2017), was achieved by the RT group in the studies by (de Lima et al. 2019; Smaili et al. 2018; Demonceau et al. 2017; Santos et al. 2017; Ferreira et al. 2018), all of which used a control group as the comparator. Additionally, the MCID was reached by the instability‐based RT group in the study by Silva‐Batista et al. (2016) and by the neurofunctional training group in the study by Smaili et al. (2018).

**FIGURE 2:**
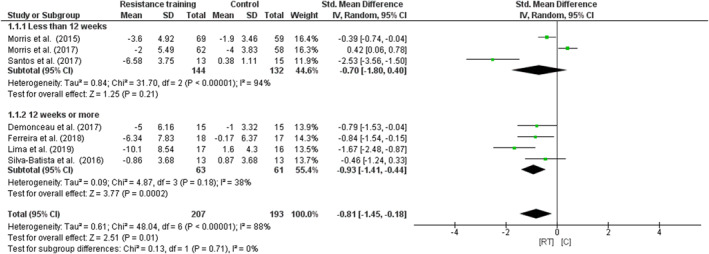
Forest plot of quality of life for resistance training versus control in people with Parkinson's disease. Subgroup analyses by intervention duration are exploratory, representing preliminary trends rather than definitive clinical prescriptions.

In the meta‐analysis, RT was compared with inactive controls and other therapeutic modalities (OTM). Seven studies evaluated QoL using the total PDQ‐39 score in comparison with a control, demonstrating a large pooled effect favoring RT, but with high statistical heterogeneity [SMD −0.81; CI −1.45 to −0.18; *I* ^2^ 88%; *p* = 0.01; RT *n* = 207, C *n* = 193; moderate certainty of the evidence] (presented in Figure 2). Therefore, this overall estimate should be interpreted cautiously. To explore potential sources of clinical heterogeneity, subgroup analyses were conducted. When stratified by intervention duration, protocols lasting less than 12 weeks did not demonstrate a significant effect, with persistent high heterogeneity [SMD −0.70; CI −1.80 to 0.40; *I* ^2^ 94%; *p* = 0.21, RT *n* = 144, C *n* = 132]. In contrast, exploratory subgroup analyses of interventions longer than 12 weeks showed a significant pooled effect and a substantial reduction in heterogeneity [SMD −0.93; CI −1.41 to −0.44; *I* ^2^ 38%; *p* = 0.0002, RT *n* = 63, C *n* = 61] (presented in Figure 2). These findings should be interpreted cautiously due to the exploratory nature of the subgroup analyses and the limited number of studies.

**FIGURE 3:**
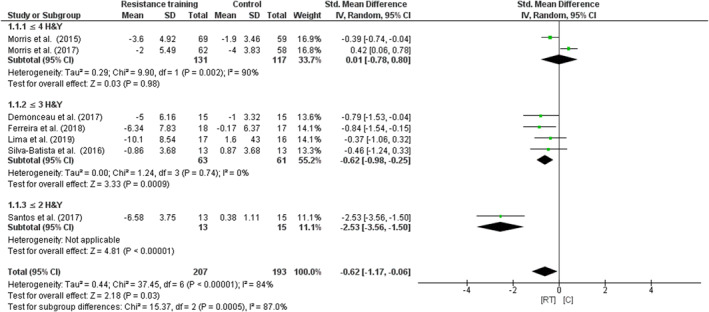
Forest plot of quality of life for resistance training versus control in people with Parkinson's disease. Subgroup analyses by disease staging (H&Y scale) are exploratory and should not be overinterpreted as definitive evidence.

**FIGURE 4:**
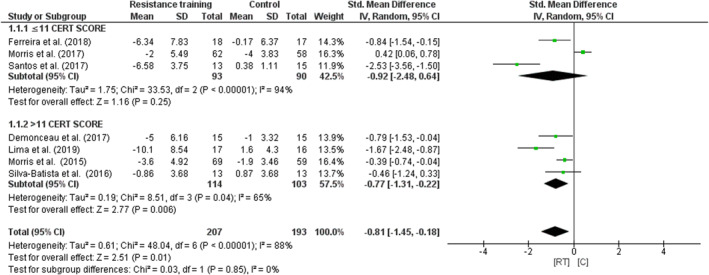
Forest plot of quality of life for resistance training versus control in people with Parkinson's disease. Subgroup analyses by CERT score are exploratory and higher scores reflect completeness of reporting and reproducibility, not treatment effectiveness.

Stratification by disease severity, based on baseline H&Y criteria, showed that studies including patients with stage ≤ 4 failed to demonstrate superiority to the control [SMD ‐0.01; CI −0.78 to 0.80; *I* ^2^ 90%; *p* = 0.98, RT *n* = 131, C *n* = 117]. However, exploratory subgroup analyses showed a significant pooled effect favoring RT in studies limiting inclusion to stages ≤ 3 [SMD −0.62; CI −0.98 to −0.25; *I* ^2^ 0%; *p* = 0.0009, RT *n* = 63, C *n* = 61] or ≤ 2, although the latter subgroup was represented by a single trial [SMD −2.53; CI −3.56 to −1.50; *p* = 0.00001, RT *n* = 13, C *n* = 15] (presented in Figure 3), noting that heterogeneity decreased exclusively within the H&Y stages ≤ 3 subgroup. Lastly, subgroup stratification by reporting completeness (CERT score) indicated that trials with more comprehensive intervention reporting (CERT > 11) showed statistically significant pooled effects favoring RT [SMD −0.77; CI −1.31 to −0.22; *I* ^2^ 65%; *p* = 0.006, RT *n* = 114, C *n* = 103], whereas those with lower reporting completeness (CERT ≤ 11) did not differ significantly from controls and exhibited high heterogeneity [SMD −0.92; CI −2.48 to 0.64; *I* ^2^ 94%; *p* = 0.25, RT *n* = 93, C *n* = 90] (presented in Figure 4). Nevertheless, these subgroup findings should be interpreted as exploratory due to the small number of studies within certain subgroups and the persistent heterogeneity across several analyses.

In the analysis comparing RT with OTM, six studies were included, utilizing the PDQ‐39 and PDQ‐8 instruments. The study by Li et al. (2014) included two OTM groups (*a* = stretching and *b* = Tai Chi), therefore, to avoid participant duplication and maintain appropriate statistical weighting, the RT sample size was adjusted. Subgroup analysis by instrument showed no significant differences favoring either intervention in the PDQ‐8 [SMD 0.32; CI −0.42 to 1.07; *I* ^2^ 89%; *p* = 0.39, RT *n* = 118, OTM *n* = 175], or the PDQ‐39 [SMD −0.24; CI −0.51 to 0.02; *I* ^2^ 0%; *p* = 0.07, RT *n* = 115, OTM *n* = 110]. Similarly, stratification by intervention duration demonstrated no significant benefits for protocols longer than 12 weeks [SMD −0.14; CI −0.73 to 0.45; *I* ^2^ 76%; *p* = 0.64, RT *n* = 88, OTM *n* = 145] or shorter than 12 weeks [SMD 0.12; CI −0.59 to 0.83; *I* ^2^ 87%; *p* = 0.74, RT *n* = 145, OTM *n* = 140]. In the overall analysis, there was also no significant pooled effect, accompanied by high heterogeneity [SMD −0.02; CI −0.44 to 0.41; *I* ^2^ 80%; *p* = 0.93, RT *n* = 233, OTM *n* = 285; low certainty of the evidence; details regarding the certainty of the evidence are available in Supporting Information S1: Section (D)] (figures presented in the Supporting Information S1: Section (E)).

A sensitivity analysis was conducted assuming a lower correlation than the true value. The overall results remained stable, with only a slight, anticipated reduction in the effect size.

A funnel plot was generated to inspect potential publication bias (Supporting Information S1: Section (F)). However, with only seven studies included in the meta‐analysis, the funnel plot has very limited interpretability. Consequently, publication bias cannot be reliably assessed in the present review, and an asymmetric distribution of studies was therefore expected.

### 3.5 ROB2 and CERT

**FIGURE 5:**
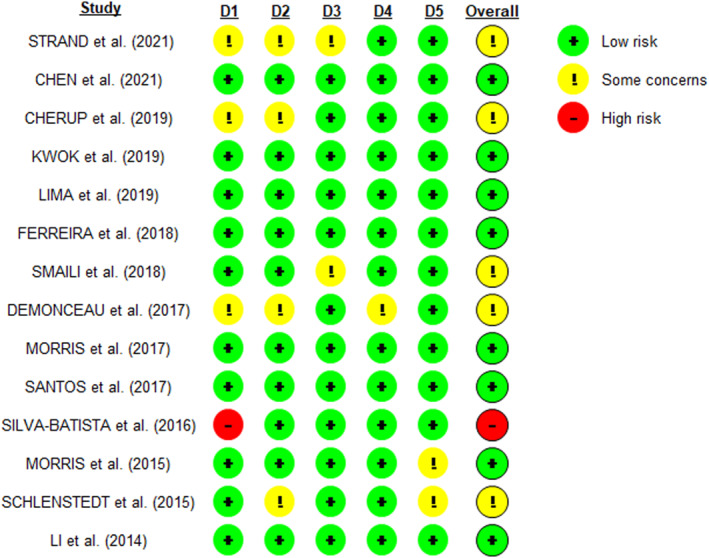
Risk of bias analysis of the studies included in the review.

The risk of bias for QoL outcome in each study was assessed using RoB2. Of the 14 included studies, one was classified as “high risk of bias” due to the allocation procedure, five were classified as having “some concerns” and eight were classified as “low risk of bias”. Given the nature of physical exercise interventions, blinding participants and personnel is not feasible; therefore, this lack of blinding is not considered as a source of bias. The individual assessment for each study is presented in Figure 5.

Regarding the completeness of the intervention descriptions assessed using the CERT checklist, a low to moderate completeness was observed, with scores ranging from 11 to 17 points. A key component absent from nearly all articles was a description of how adherence was measured. The detailed CERT scores for each study are provided in Supporting Information S1: Section (G).

## 4 Discussion

This systematic review suggests that RT may improve QoL in individuals with PD. However, RT was not found to be superior to other physical exercise interventions. These findings should be interpreted with caution given the substantial heterogeneity across study protocols and analyses. The certainty of evidence was moderate for comparisons with control groups and low for comparisons with other physical exercise modalities.

QoL was assessed using the 8‐domain PDQ‐39 and PDQ‐8 questionnaires (K. Chen et al. 2017; Jenkinson et al. 1997). Among the three studies that reported all domains (J. Chen et al. 2021; Smaili et al. 2018; Demonceau et al. 2017), two demonstrated significant improvements in the mobility, well‐being, and activities of daily living domains. Because RT is known to improve muscle strength, it is plausible that the physical domains of QoL are more responsive to this intervention. Additionally, RT is effective in reducing depression and anxiety in several populations (Andrade et al. 2019; Vilarino et al. 2023, 2021), which is consistent with the significant improvements observed in some studies within the emotional well‐being domain (J. Chen et al. 2021; Smaili et al. 2018).

Most studies evaluated interventions lasting 12 weeks or more, whereas five protocols ranged from 6 to 8 weeks (Morris et al. 2015, 2017; Kwok et al. 2019; Santos et al. 2017; Schlenstedt et al. 2015). Exploratory subgroup analyses suggested a potential trend toward more favorable QoL outcomes in studies lasting longer than 12 weeks; however, this finding remains exploratory and is characterized by substantial heterogeneity across protocols. In the trial by Corcos et al. (2013), QoL improvements were no longer significant after 24 months of training. This observation may suggest that periodic adjustments to exercise protocols could help reduce training plateaus, although further research is required. In addition, positive findings were more frequently observed in studies implementing two weekly sessions; however, these observations remain exploratory and may be influenced by other protocol characteristics, such as intervention duration, disease severity, and training intensity. Three studies performed RT once a week (Morris et al. 2015, 2017; Kwok et al. 2019), and reported no significant improvements. However, these protocols also had short durations of 6 weeks (Morris et al. 2017) and 8 weeks (Morris et al. 2015; Kwok et al. 2019), making it difficult to isolate the independent influence of training frequency from that of intervention duration.

Prescribed intensities presented high heterogeneity, limiting direct protocol comparison; this limitation is consistent with previous systematic reviews that reported similar challenges (Brienesse and Emerson 2013; X. Li et al. 2020). Progression strategies varied widely, including 1‐RM percentages (Ferreira et al. 2018; Lima et al. 2013), rating of perception of exertion (Morris et al. 2015), and pre‐specified load or volume adjustments (Demonceau et al. 2017; Santos et al. 2017); some studies did not report progression strategies (Morris et al. 2017). However, training volumes were relatively consistent across trials, typically comprising 2–3 sets of 8–15 repetitions, in accordance with established RT recommendations (Garber et al. 2011).

Regarding the RT protocols, interventions varied from machine‐based exercises (J. Chen et al. 2021; Strand et al. 2021; Cherup et al. 2019; Santos et al. 2017) to combinations of machines, free weights, or body weight (de Lima et al. 2019; Ferreira et al. 2018; Smaili et al. 2018; Morris et al. 2015; Kwok et al. 2019; Demonceau et al. 2017; Schlenstedt et al. 2015). However, the available studies did not demonstrate a clear advantage of one equipment modality over another, as various protocols improved QoL without showing clear superiority. These findings may suggest that traditional gym infrastructure is not always required to implement RT programs; however, the available evidence remains insufficient to determine whether equipment type influences outcomes. The studies conducted by (Morris et al. 2015, 2017; Schlenstedt et al. 2015) that used only bodyweight and elastic bands found no significant benefits; however, these results are likely confounded by their short intervention durations of less than 12 weeks.

To explore clinical diversity, subgroup analyses were performed stratified by intervention duration (weeks) and applied the CERT to evaluate the completeness of exercise intervention reporting. However, because these analyses are exploratory and involve considerable clinical and statistical heterogeneity, definitive parameters for specific clinical prescriptions cannot be established. Consequently, the overall pooled effect should be interpreted as a “class effect” of RT on QoL rather than as an endorsement of any single specific protocol.

When compared with other physical exercise modalities, RT did not demonstrate significant differences in QoL outcomes (Strand et al. 2021; Smaili et al. 2018; Cherup et al. 2019; Morris et al. 2017; Schlenstedt et al. 2015). Furthermore, the certainty of evidence was low due to substantial heterogeneity and wide confidence intervals, reinforcing the need for additional high‐quality trials. However, some original trials reported significant differences favoring mindfulness yoga and Tai Chi over RT (F. Li et al. 2014; Kwok et al. 2019). In contrast, pooled analyses comparing RT with inactive control groups favored RT, with moderate certainty of evidence downgraded due to heterogeneity. A recent systematic review with meta‐analysis evaluating different exercise modalities for individuals with PD similarly reported no clear superiority one exercise type over another, although exercise interventions generally demonstrated benefits compared with non‐intervention groups (Ernst et al. 2023). Collectively, these findings suggest that physical exercise may contribute to improvements in QoL in individuals with PD, regardless of the specific modality implemented, consistent with current World Health Organization recommendations (Bull et al. 2020).

Furthermore, the present review explored two additional factors that have received limited attention in previous reviews. First, studies with more comprehensively reported exercise protocols (CERT > 11) tended to show more consistent findings, suggesting the importance of adequate intervention reporting for reproducibility and clinical interpretation. Importantly, CERT evaluates reporting completeness and reproducibility rather than intervention effectiveness. Therefore, these findings should not be interpreted as evidence that better‐reported interventions are inherently more effective. Second, baseline disease severity may represent a relevant factor, as studies including participants in H&Y stages ≤ 3 more frequently reported significant QoL improvements. This finding may be related to the greater functional capacity typically observed in earlier disease stages (Petzinger et al. 2010). However, these observations remain exploratory and should be interpreted cautiously due to the limited number of studies and the substantial heterogeneity across interventions.

Because PD is a progressive neurodegenerative disease, individuals typically experience a worsening of symptoms over time (Poewe and Mahlknecht 2009), RT may assist individuals in managing disease progression by enhancing cognitive and motor function, mitigating symptom progression (Pinho et al. 2019; Herold et al. 2019), and improving QoL.

Although previous systematic reviews have examined exercise interventions and RT in PD, this review extends the current evidence by incorporating intervention reporting completeness and disease‐stage subgroup analyses in relation to QoL outcomes. Several limitations must be acknowledged. Although most studies were classified as having low risk of bias or some concerns, such variation is common but not ideal in exercise trials. The evidence is constrained by overlapping samples, as two research groups were responsible for four of the studies analyzed (de Lima et al. 2019; Ferreira et al. 2018; Morris et al. 2015, 2017). The included studies exhibited substantial heterogeneity regarding exercise intensity prescription and intervention duration. It must also be acknowledged that some subgroup analyses were based on a small number of studies, or even a single trial, limiting the robustness of these findings. Therefore, the subgroup results should be interpreted as exploratory and hypothesis‐generating rather than as evidence supporting specific prescription parameters. Additionally, the imputation of standard deviations for mean differences, necessitated by the absence of original data, should be considered a methodological limitation when interpreting the findings. Future high‐quality randomized trials are needed to evaluate the effects of RT on QoL in this population, using well‐established protocols and comparing different RT protocols. Future studies should also standardize the methods of quantifying and reporting training intensity, given that this variable presented the greatest heterogeneity. Finally, subsequent clinical trials should also include individuals in advanced stages of PD and clearly report disease staging at baseline, thereby contributing to more informed clinical decision‐making.

This review extends the existing literature by summarizing the evidence on the effects of RT and QoL in PD, while also exploring intervention reporting completeness and disease‐stage subgroup analyses. These findings may be applied by health professionals across different clinical contexts, supporting the consideration of RT as a complementary therapeutic option.

In conclusion, the available evidence suggests that RT may be a useful therapeutic option for improving QoL in individuals with PD and appears to be safe. The certainty of evidence is moderate when RT is compared to control, and low when compared to other exercise modalities, which offer similar benefits. However, the high clinical and statistical heterogeneity observed across protocols limits the ability to draw definitive conclusions or establish specific prescription parameters. While improvements were frequently observed in interventions lasting over 12 weeks, with a frequency of 2–3 sessions per week (40–60 min), using various resistance modalities (free weights, resistance bands, or machines), these trends should be interpreted strictly as tentative observations rather than as clinical prescriptions. Furthermore, exploratory subgroup analyses suggested more favorable results in studies limited to participants with H&Y ≤ 3; however, this observation requires confirmation in future trials. Given that most studies included in this review provided incomplete documentation of their protocols, improving reporting completeness according to CERT recommendations may enhance reproducibility and facilitate the interpretation of future findings. Ultimately, the high overall heterogeneity and substantial variability in duration, intensity, and progression methods across the current literature remain defining limitations, requiring a highly cautious interpretation of the pooled findings.

## 5 Implications for Physiotherapy Practice

This systematic review suggests that RT may contribute to improvements in QoL in individuals with PD. Exploratory analyses suggested that studies implementing RT protocols lasting longer than 12 weeks with 2–3 weekly sessions more frequently reported QoL improvements. However, due to high clinical and statistical heterogeneity, these parameters represent preliminary trends from exploratory analyses and should not be interpreted as prescriptive guidelines. To mitigate training plateaus, physiotherapists should consider incorporating progression strategies based on ratings of perceived exertion or 1‐RM assessments. Resistance can be applied using machines, free weights, or elastic bands, allowing the interventions to be tailored to the patient's context and easily implemented in resource‐limited settings.

These exploratory findings apply primarily to individuals with PD in H&Y stages ≤ 3, however, data categorized by disease stage remain limited. RT appears to be a safe modality; nonetheless, clinicians should remain vigilant for joint pain. Professionals must prioritize trials with high reporting quality to ensure reproducibility, integrating evidence‐based practice by opting for well‐reported interventions with explicit details (e.g., following CERT).

Finally, QoL represents an important clinical outcome for evaluation and monitoring in individuals with PD. Overall, RT appears to be a feasible therapeutic option that may be integrated with other evidence‐based exercise modalities according to individual characteristics and patient preferences.

## Author Contributions

**Loiane Cristina de Souza:** conceptualization, data curation, formal analysis, methodology, writing – original draft, writing – review and editing, visualization. **Luiz Senna:** conceptualization, methodology, investigation, data curation, formal analysis, writing – original draft. **Anderson D'Oliveira:** software, methodology, investigation. **Rubia Truppel:** software, investigation, formal analysis, methodology. **Guilherme Torres Vilarino:** writing – review and editing, methodology, investigation. **Alexandro Andrade:** writing – review and editing, visualization, supervision.

## Ethics Statement

This study is a systematic review and meta‐analysis of previously published data. As such, no new data were collected directly from human participants, and informed consent was not applicable. All included studies in the analysis were conducted in compliance with ethical standards and obtained appropriate informed consent from their participants as stated in their respective publications. The authors confirm that the approval of an institutional review board was not required for this work.

## Consent

The authors have nothing to report.

## Conflicts of Interest

The authors declare no conflicts of interest.

## Supporting information


Supporting Information S1

## Data Availability

Data sharing not applicable to this article as no datasets were generated or analysed during the current study.
