# Supplementary material for: Does Resistance Training Improve the Quality of Life of People With Parkinson's Disease? Evidence and Recommendations for Clinical Application Through a Systematic Review and Meta‐Analysis of Randomized Clinical Trials
Source: Physiother Res Int. 2026 Jun 29;31(3):e70266. doi: 10.1002/pri.70266 (PMC13312437; doi:10.1002/pri.70266)
Supplement: Supplementary file 1 — Supporting Information S1 [file PRI-31-e70266-s001.docx]

Supplementary Material List

| A | Complete search strategy in all databases |
| --- | --- |
| B | Table of results of all individual studies |
| C | Table with mean and standard deviation values of quality of life before and after RT intervention in patients with Parkinson's disease |
| D | Certainty of evidence - GRADE |
| E | Forest plot RT x OTM |
| F | Funnel Plot |
| G | CERT Scores for each study |

**A - Complete search strategy in all databases**

| **Search Details - PUBMED** |
| --- |
| #1 AND #2 |
| "Muscle Strengthening"[Title/Abstract] OR (("resist"[All Fields] OR "resistance"[All Fields] OR "resistances"[All Fields] OR "resistant"[All Fields] OR "resistants"[All Fields] OR "resisted"[All Fields] OR "resistence"[All Fields] OR "resistence"[All Fields] OR "resisting"[All Fields] OR "resistibility"[All Fields] OR "resisting"[All Fields] OR "resistive"[All Fields] OR "resistively"[All Fields] OR "resistivities"[All Fields] OR "resistivity"[All Fields] OR "resists"[All Fields]) AND "exercise"[MeSH Terms]) OR "resistance training"[MeSH Terms] OR "resistance training"[MeSH Terms] |
| "parkinson disease"[MeSH Terms] OR "Parkinson"[Title/Abstract] OR "Parkinsonian"[Title/Abstract] OR "parkinson's"[Title/Abstract] OR "parkinsons"[Title/Abstract] |
| **Search Details – WOS** |
| (((TS=("resistance training")) OR TS=("strength training")) OR TS=("Resistance Exercise")) OR TS=("Muscle Strengthening") **AND** ((((TS=("Parkinson disease")) OR TS=("Parkinson")) OR TS=("Parkinsonian")) OR TS=("parkinson's")) OR TS=("parkinsons") |
| **Search Details – EMBASE** |
| ('parkinson disease':ab,ti OR 'parkinson':ab,ti OR 'parkinsonian':ab,ti OR 'parkinsons':ab,ti) AND ('resistance training':ab,ti OR 'strength training':ab,ti OR 'resistance exercise':ab,ti OR 'muscle strengthening':ab,ti) |
| **Search Details – SCOPUS** |
| ( ( TITLE-ABS ( "Muscle Strengthening" ) ) OR ( TITLE-ABS ( "Resistance Exercise" ) ) OR ( TITLE-ABS ( "strength training" ) ) OR ( TITLE-ABS ( "resistance training" ) ) ) AND ( ( TITLE-ABS ( "parkinson disease" ) ) OR ( TITLE-ABS ( "parkinson" ) ) OR ( TITLE-ABS ( "Parkinsonian" ) ) OR ( TITLE-ABS ( "parkinson&apos;s" ) ) OR ( TITLE-ABS ( "parkinsons" ) ) ) |
| **Search Details – CINAHL** |
| TI (("Parkinson disease" OR Parkinsonian OR Parkinson OR “parkinson’s” OR parkinsons) AND TI ((“Resistance Training” OR “Strength Training” OR “Resistance Exercise” OR “Muscle Strengthening”) |
| **Search Details – PEDro** |
| Parkinson* AND "resistance training" (filter: clinical trials) |

| **B - Table of results of all individual studies** | | | | | | | |
| --- | --- | --- | --- | --- | --- | --- | --- |
| Reference | Country | Total Sample N | Adverse events | Adherence and sample withdrawal | Intragroup difference (pre and post) | Difference with control group | Difference with another exercise |
| STRAND et al. (2021) | United States | 35 | Discomfort while performing the chest press exercise (required no medical attention). | **SPHG: 4** withdrawals  **SPG+Func: 3** withdrawals  "2 were excluded for attending less than 75% of classes" | **SPHG:** No significant difference  **SPG+Func:** No significant difference | It does not have a CG | No significant difference between the groups |
| CHEN et al. (2021) | Brazil | 74 | **RTFWG:** fall episode (one participant), mild transient joint pain (three participants) and orthostatic hypotension (three participants)  **RTMG**: outdoor fall (one participant),  mild transient muscle pain (three participants) and orthostatic hypotension (two participants).  **CG**: joint pain (one participant). | **RTMG:** 1 withdrawal at 3 months **and** 2 withdrawals at 6 months.  **RTFWG: 2** withdrawals at 3 months **/ 6** withdrawals at 6 months  **CG: 3** withdrawals at 3 months **/ 4** withdrawals at 6 months | **RTMG:** PDQ-mobility (p=0.001)  **RTFWG:** No significant difference  **CG:** No significant difference | A significant interaction was observed in the group-time analysis for the mobility domain of the PDQ-39 (p=0.019), not specified at which time or group | |
| CHERUP et al. (2019) | United States | 35 | Adverse events were not assessed. | **STG:** 3 withdrawals  **PTG:** 4 withdrawals  “Both training protocols were well-tolerated with  the 35 participants attending an average 22 out of 24 sessions (92.6% adherence).” | **STG:** The total score showed a significant difference after 12 weeks (p = 0.029).  **PTG:** There was no significant difference in total score after 12 weeks  The analysis of the PDQ-39 components showed significant group-time interaction (p=0.042) | It does not have a CG | No significant difference between the groups |
| KWOK et al. (2019) | China | 138 | **YMG:** knee pain (three participants)  **RTSG:** knee pain during squads (two participants) | **YMG:** 13 withdrawals at 8 weeks/ 14 withdrawals at 20 weeks  **RTSG:** 8 withdrawals at 8 weeks **/** 12 withdrawals at 20 weeks**)** | **YMG:** No significant difference  **RTSG:** No significant difference | It does not have a CG | This was a significant difference (p=0.001), with **GYM** presenting a lower score. |
| LIMA et al. (2019) | Brazil | 33 | Adverse events were not assessed. | No data | **RTG:** there was a significant difference after 20 weeks  **CG:** showed no significant differences after 20 weeks | There was a significant difference for groupxtime interaction (p = 0.007), and the **TGR** had a lower score in the post-period (**GTR** 30.2 and **CG** 40.6) | It did not present another group |
| FERREIRA et al. (2018) | Brazil | 35 | No adverse events. | There were no dropouts. | **RTG:** significant difference (p=0.009)  **CG:** No difference | Not analyzed | It did not present another group |
| SMAILI et al. (2018) | Brazil | 40 | Adverse events were not assessed. | **RTG:** 5 withdrawals  **NTG:** 9 withdrawals | **RTG:** PDQ-total (p=0.003); PDQ-mobility (p=0.006); PDQ-life activities (p=0.01) and PDQ-well-being (p=0.02).  **NTG:** PDQ-total (p=0.02); PDQ-activities life (p=0.04) and PDQ-well-being (p=0.04) | It does not have a CG | No significant difference |
| DEMONCEAU et al. (2017) | Belgium | 52 | **STG:** Excessive tiredness, Patella pain syndrome (2 participants), back pain, Reappearance of pain linked to an previous elbow fracture, Exacerbation of pain from a previous wrist sprain .  **ATG:** Light knee sprain, knee pain, Headache, Tiredness, Effort persisting hypotension. | **STG:** 2 withdrawals. Mean of 31±5.5 sessions per patients.  **ATG:** 4 withdrawals. Mean of 31.3±5.5 total sessions per patient. | There was a significant effect of "time" between the groups for the *PDQ-well-being* (p=0.034), where the **STG** group showed an improvement of -37% in the score and effect size of Cohen's d of -0.57.  There was no significance in the post hoc analysis. | Significant groupxtime interaction in the *PDQ-stigma* (p<0.05), where the **STG** (-26%) and **ATG** (-30%) had a reduction in the score while the **CG** (+27%) had an increase.  There was a significant difference between groups in the *PDQ-social support* (p=0.012), where the **ATG** group showed a 32% reduction in the score (**STG** and **CG** without reduction).  There was no significance in the post hoc analysis | |
| MORRIS et al. (2017) | Australia | 133 | No adverse events. | **RTG:** 5 withdrawals  **CG:** 8 withdrawals  “Adherence to the unsupervised sessions was high, with 62 of  experimental group and 51 of control group participants receiving  5 to 6 weeks of therapy.” | **RTG:** No significant difference  **CG:** No difference No significant difference | No significant difference between the groups | It did not present another group |
| SANTOS et al. (2017) | Spain | 28 | No adverse events. | There were no dropouts.  *Participants were required to complete a minimum of 15 of the 16 scheduled training sessions (representing >90% attendance) to be included in data analysis. | **RTG:** significant difference (p=0.024)  **CG:** No significant difference | Significant difference after intervention (*p=0.000), with the **RTG** presenting a lower score | It did not present another group |
| SILVA-BATISTA et al. (2016) | Brazil | 39 | No adverse events. | There were no dropouts.  “Adherence to the protocol was high for both training groups  (98% for RTG and 97% for RTIG”. | **RTG:** No significant difference  **RTIG:** PDQ-39 (p<0.001)  **CG:** No significant difference | Group*x*time interaction (p<0.0001), not specifying which group.  In the post hoc analysis, there were no significant differences between the groups. | |
| MORRIS et al. (2015) | Australia | 210 | No adverse events. | **MSTG:** 1 withdrawal  **CG**: 5 withdrawals  “Adherence to all sessions was excellent, with  90% of participants attending 6 or more of the 8 scheduled  sessions. Participant attendance (as defined by attendance at  ≥6 sessions) did not differ across the 3 groups” | **RTG:** No significant difference  **MSTG:** No significant difference **CG:** No significant difference | Not analyzed | Not analyzed |
| SCHLENSTEDT et al. (2015) | Germany | 40 | They only report that the dropouts did not occur due to the protocols, but do not detail whether there were adverse events. | **RTG: 8** withdrawals  **BG: 5** withdrawals | **RTG:** No significant difference  **BG:** No significant difference | It does not have a CG | No significant difference between the groups |
| LI et al. (2014) | United States | 195 | No adverse events. | **RTG: 6** withdrawals  **TCG: 9** withdrawals  **SG: 4** withdrawals | **RTG:** significant difference (p<0.01);  **TCG:** significant difference (p<0.01);  **SG:** No significant difference | It does not have a CG | Significant difference for **GTC** in relation to **GTR** (p=0.014) and **GA** (P<0.001). |

**Legend:** SPHG: strength, power and hypertrophy group; SPG+Func: strength and power group with functional training; CG: control group; RTMG: resistance training in machine group; RTFWG: resistance training with free weight group; STG: strength training group; PTG: power training group; YMG: yoga mindfulness group; RTSG: resistance training and stretching group; RTG: resistance training group; NTG: neurofunctional training group; ATG: aerobic training group; RTIG: resistance training with instability group; MSTG: movement strategy training group; BG: balance training group; TCG: Tai Chi group; SG: stretching group.

| **C - Table of mean and standard deviation values of quality of life before and after the intervention of RT in patients with Parkinson's disease** | | | | | | | | | |
| --- | --- | --- | --- | --- | --- | --- | --- | --- | --- |
| **Reference** | **Instrument** | **Groups** | **N-pre** | **N-post** | **Pre Mean** | **Pre SD** | **Post Mean** | **Post SD** | **Mean difference** |
| STRAND et al. (2021) | PDQ-39 | SPHG | 17 | 13 | 21.69 | 14.20 | 18.80 | 11.05 | -2,89 |
|  |  | SPG+Func | 18 | 15 | 16.91 | 8.64 | 14.57 | 8.38 | -2,34 |
| CHEN et al. (2021) | PDQ-39 - Mobility | RTMG | 23 | 23 | 34.72 | 24.10 | 21.46 | 21.04 | -13,26 |
|  |  | RTFWG | 26 | 26 | 30.52 | 22.02 | 23.94 | 19.34 | -6,58 |
|  |  | CG | 25 | 25 | 23.12 | 19.51 | 24.94 | 17.64 | 1,82 |
|  | PDQ-39 – Daily Life | RTMG | 23 | 23 | 37.58 | 23.70 | 25.21 | 19.53 | -12,37 |
|  |  | RTFWG | 26 | 26 | 36.53 | 25.99 | 28.21 | 21.14 | -8,32 |
|  |  | CG | 25 | 25 | 24.24 | 21.44 | 24.03 | 16.41 | -0,21 |
|  | PDQ-39 – Emotional Well-Being | RTMG | 23 | 23 | 30.79 | 22.68 | 21.04 | 16.54 | -9,75 |
|  |  | RTFWG | 26 | 26 | 28.54 | 23.44 | 22.61 | 15.08 | -5,93 |
|  |  | CG | 25 | 25 | 22.36 | 16.55 | 22.68 | 15.42 | 0,32 |
|  | PDQ-39 - Stigma | RTMG | 23 | 23 | 20.90 | 21.62 | 19.03 | 22.65 | -1,87 |
|  |  | RTFWG | 26 | 26 | 25.03 | 18.38 | 19.73 | 18.17 | -5,3 |
|  |  | CG | 25 | 25 | 12.78 | 15.89 | 13.49 | 18.46 | 0,71 |
|  | PDQ-39 – Social Support | RTMG | 23 | 23 | 15.78 | 16.84 | 9.06 | 13.68 | -6,72 |
|  |  | RTFWG | 26 | 26 | 15.88 | 22.87 | 11.54 | 22.74 | -4,34 |
|  |  | CG | 25 | 25 | 8.19 | 13.95 | 9.33 | 15.07 | 1,14 |
|  | PDQ-39 - Cognition | RTMG | 23 | 23 | 26.50 | 20.87 | 25.30 | 17.55 | -1,2 |
|  |  | RTFWG | 26 | 26 | 27.44 | 20.35 | 27.39 | 19.60 | -0,05 |
|  |  | CG | 25 | 25 | 19.52 | 19.30 | 18.78 | 15.62 | -0,74 |
|  | PDQ-39 - Communication | RTMG | 23 | 23 | 26.19 | 20.64 | 22.49 | 19.56 | -3,7 |
|  |  | RTFWG | 26 | 26 | 26.28 | 26.22 | 23.65 | 19.49 | -2,63 |
|  |  | CG | 25 | 25 | 27.49 | 17.99 | 23.97 | 16.36 | -3,52 |
|  | PDQ-39 – Physical discomfort | RTMG | 23 | 23 | 42.43 | 24.66 | 26.78 | 22.02 | -15,65 |
|  |  | RTFWG | 26 | 26 | 25.96 | 26.01 | 25.47 | 14.05 | -0,49 |
|  |  | CG | 25 | 25 | 34.97 | 21.89 | 32.32 | 20.87 | -2,65 |
| CHERUP et al. (2019) | PDQ-39 | STG | 21 | 18 | NR | NR | NR | NR | NR |
|  |  | PTG | 21 | 17 | NR | NR | NR | NR | NR |
| KWOK et al. (2019) | PDQ-8 | YMG | 71 | 58 | 9.79 | 5.02 | 7.57 | 4.68 | -2,22 |
|  |  | RTSG | 67 | 59 | 9.21 | 5.26 | 9.66 | 5.05 | 0,45 |
| LIMA et al. (2019) | PDQ-39 | RTG | 17 | 17 | 40.3 | 21.1 | 30.2 | 16.8 | -10,1 |
|  |  | CG | 16 | 16 | 39 | 16.8 | 40.6 | 15.6 | 1,6 |
| FERREIRA et al. (2018) | PDQ-39 | RTG | 18 | 18 | 38.06 | 20.16 | 31.72 | 19.75 | -6,34 |
|  |  | CG | 17 | 17 | 47.41 | 17.27 | 47.24 | 21.34 | -0,17 |
| SMAILI et al. (2018) | PDQ-39 – Total | RTG | 19 | 14 | 27.4 | 12.4 | 19.1 | 10.9 | -8,3 |
|  |  | NTG | 21 | 12 | 27.2 | 17.7 | 22.3 | 17.7 | -4,9 |
|  | PDQ-39 - Mobility | RTG | 19 | 14 | 28.3 | 23.3 | 19.5 | 19.5 | -8,8 |
|  |  | NTG | 21 | 12 | 26.0 | 24.4 | 21.3 | 25.3 | -4,7 |
|  | PDQ-39 – Activities of Daily Living | RTG | 19 | 14 | 35.5 | 24.3 | 24.3 | 18.0 | -11,2 |
|  |  | NTG | 21 | 12 | 33.3 | 29.3 | 24.7 | 25.0 | -8,6 |
|  | PDQ-39 – Emotional Well-Being | RTG | 19 | 14 | 30.3 | 19.2 | 22.0 | 22.0 | -8,3 |
|  |  | NTG | 21 | 12 | 29.1 | 21.5 | 23.5 | 20.8 | -5,6 |
|  | PDQ-39 - Stigma | RTG | 19 | 14 | 8.5 | 10.8 | 7.3 | 10.4 | -1,2 |
|  |  | NTG | 21 | 12 | 14.3 | 12.6 | 12.5 | 13.4 | -1,8 |
|  | PDQ-39 – Social Support | RTG | 19 | 14 | 12.2 | 18.6 | 6.3 | 17.5 | -5,9 |
|  |  | NTG | 21 | 12 | 7.3 | 17.1 | 4.9 | 11.4 | -2,4 |
|  | PDQ-39 - Cognition | RTG | 19 | 14 | 31.2 | 22.7 | 11.2 | 15.5 | -20 |
|  |  | NTG | 21 | 12 | 39.3 | 25.4 | 34.1 | 27.2 | -5,2 |
|  | PDQ-39 - Communication | RTG | 19 | 14 | 22.0 | 25.1 | 11.2 | 15.5 | -10,8 |
|  |  | NTG | 21 | 12 | 24.0 | 21.0 | 23.0 | 21.5 | -1 |
|  | PDQ-39 – Physical discomfort | RTG | 19 | 14 | 43.8 | 20.3 | 35.7 | 20.5 | -8,1 |
|  |  | NTG | 21 | 12 | 39.2 | 34.2 | 33.3 | 29.6 | -5,9 |
| DEMONCEAU et al. (2017) | PDQ-39 – Total | ATG | 20 | 16 | 28 | 12 | 27 | 15 | -1 |
|  |  | STG | 17 | 15 | 24 | 12 | 19 | 7 | -5 |
|  |  | CG | 15 | 15 | 20 | 13 | 19 | 13 | -1 |
|  | PDQ-39 - Mobility | ATG | 20 | 16 | 10 | 0-22,5 | 11.25 | 3.75-30 | -1,25 |
|  |  | STG | 17 | 15 | 15 | 7.5-20 | 15 | 7.5-22.5 | 0 |
|  |  | CG | 15 | 15 | 10 | 0-22.5 | 7.5 | 0-20 | -2,5 |
|  | PDQ-39 – Activities of Daily Living | ATG | 20 | 16 | 30 | 18 | 29 | 19 | -1 |
|  |  | STG | 17 | 15 | 26 | 21 | 24 | 17 | -2 |
|  |  | CG | 15 | 15 | 25 | 17 | 23 | 15 | -2 |
|  | PDQ-39 – Emotional Well-Being | ATG | 20 | 16 | 33 | 16 | 29 | 19 | -4 |
|  |  | STG | 17 | 15 | 22 | 16 | 14 | 11 | -8 |
|  |  | CG | 15 | 15 | 21 | 22 | 19 | 21 | -2 |
|  | PDQ-39 - Stigma | ATG | 20 | 16 | 28 | 23 | 19 | 19 | -9 |
|  |  | STG | 17 | 15 | 23 | 18 | 17 | 14 | -6 |
|  |  | CG | 15 | 15 | 11 | 12 | 14 | 15 | 3 |
|  | PDQ-39 – Social Support | ATG | 20 | 16 | 25 | 0-33 | 17 | 0-29 | -8 |
|  |  | STG | 17 | 15 | 0 | 0-17 | 0 | 0-0 | 0 |
|  |  | CG | 15 | 15 | 0 | 0-17 | 0 | 0-25 | 0 |
|  | PDQ-39 - Cognition | ATG | 20 | 16 | 35 | 24 | 34 | 26 | -1 |
|  |  | STG | 17 | 15 | 30 | 22 | 24 | 20 | -6 |
|  |  | CG | 15 | 15 | 27 | 18 | 25 | 18 | -2 |
|  | PDQ-39 - Communication | ATG | 20 | 16 | 33 | 12-42 | 29 | 16-37 | -4 |
|  |  | STG | 17 | 15 | 42 | 16-50 | 25 | 0-58 | -17 |
|  |  | CG | 15 | 15 | 25 | 0-33 | 25 | 8-33 | 0 |
|  | PDQ-39 – Physical discomfort | ATG | 20 | 16 | 46 | 23 | 46 | 26 | 0 |
|  |  | STG | 17 | 15 | 42 | 20 | 33 | 15 | -9 |
|  |  | CG | 15 | 15 | 37 | 19 | 33 | 21 | -4 |
| MORRIS et al. (2017) | PDQ-39 | RTG | 67 | 62 | 23 | 14 | 21 | 14 | -2 |
|  |  | CG | 66 | 58 | 24 | 15 | 20 | 14 | -4 |
| SANTOS et al. (2017) | PDQ-39 | RTG | 13 | 13 | 11.16 | 7.39 | 4.58 | 4.37 | -6,58 |
|  |  | CG | 15 | 15 | 10.60 | 4.38 | 10.98 | 4.32 | 0,38 |
| SILVA-BATISTA et al. (2016) | PDQ-39 | RTG | 13 | 13 | 41.3 | 9.5 | NR | NR | -1.2 |
|  |  | RTIG | 13 | 13 | 40.4 | 10.8 | NR | NR | -5.2 |
|  |  | CG | 13 | 13 | 41.8 | 14.5 | NR | NR | 0.7 |
| MORRIS et al. (2015) | PDQ-39 | RTG | 70 | 69 | 22.1 | 12.5 | 18.5 | 12.6 | -3,6 |
|  |  | MSTG | 69 | 67 | 19.4 | 12.8 | 16.9 | 14 | -2,5 |
|  |  | CG | 71 | 59 | 20.8 | 13.6 | 18.9 | 13.5 | -1,9 |
| SCHLENSTEDT et al. (2015) | PDQ-39 | RTG | 20 | 17 | 28.5 | 12.7 | 26.5 | 12.0 | -2 |
|  |  | BG | 20 | 15 | 28.5 | 17.7 | 30.2 | 17.8 | 1,7 |
| LI et al. (2014) | PDQ-8 | RTG | 65 | 59 | 25.28 | 14.97 | 21.39 | 12.72 | -3,89 |
|  |  | TCG | 65 | 56 | 25.14 | 16.81 | 15.48 | 11.35 | -9,66 |
|  |  | SG | 65 | 61 | 25.19 | 16.27 | 25.10 | 15.55 | -0,09 |

**Legend:** *In green are the results that reached the Minimal clinically important difference according to Horváth (2017) of -4.72 (PDQ-39) and -5,94 (PDQ-8)^[[1]](#footnote-1)^.* N: number os participants; PDQ-39: Parkinson's Disease Questionnaire-39; PDQ-8: Parkinson's Disease Questionnaire-8 items; SD; standart deviation; SPHG: strength, power and hypertrophy group; SPG+Func: strength and power group with functional training; CG: control group; RTMG: resistance training in machine group; RTFWG: resistance training with free weight group; STG: strength training group; PTG: power training group; YMG: yoga mindfulness group; RTSG: resistance training and stretching group; RTG: resistance training group; NTG: neurofunctional training group; ATG: aerobic training group; RTIG: resistance training with instability group; MSTG: movement strategy training group; BG: balance training group; TCG: Tai Chi group; SG: stretching group

**D – Certainty of evidence - GRADE**

**Question:** RT compared to OTM for QoL in Parkinson

| **Certainty assessment** | | | | | | | **№ of patients** | | **Effect** | | **Certainty** |
| --- | --- | --- | --- | --- | --- | --- | --- | --- | --- | --- | --- |
| **№ of studies** | **Study design** | **Risk of bias** | **Inconsistency** | **Indirectness** | **Imprecision** | **Other considerations** | **RT** | **OTM** | **Relative (95% CI)** | **Absolute (95% CI)** |  |
| 6 | randomised trials | not serious | serious | not serious | serious | none | 233 | 285 | - | SMD -**0.02 SD lower** (-0.44 to 0.41) | ⨁⨁◯◯ Low |

**CI:** confidence interval; **SMD:** standardised mean difference

**Question:** RT compared to Control for QoL in Parkinson

| **Certainty assessment** | | | | | | | **№ of patients** | | **Effect** | | **Certainty** |
| --- | --- | --- | --- | --- | --- | --- | --- | --- | --- | --- | --- |
| **№ of studies** | **Study design** | **Risk of bias** | **Inconsistency** | **Indirectness** | **Imprecision** | **Other considerations** | **RT** | **Control** | **Relative (95% CI)** | **Absolute (95% CI)** |  |
| 7 | randomised trials | not serious | very serious | not serious | serious^a^ | strong association dose response gradient | 207 | 193 | - | SMD -**0.81 SD lower** (-1.45 to - 0.18) | ⨁⨁⨁◯ Moderate^a^ |

**CI:** confidence interval; **SMD:** standardised mean difference

**E – Forest plot RT x OTM**

**D.1 Subgroup analyses by instrument**


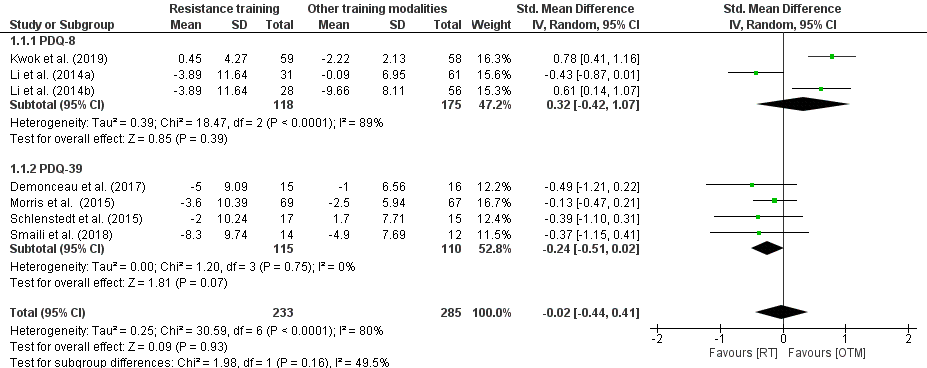


**D.2 Subgroup analyses by duration of the intervention**


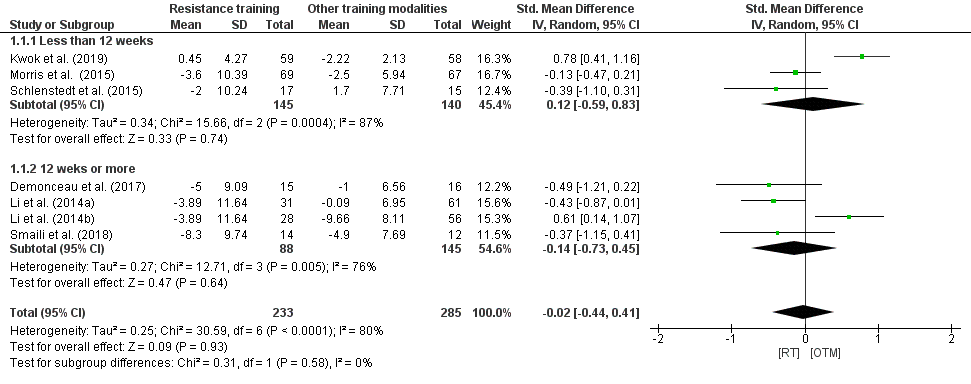


**F – Funnel plot**

**
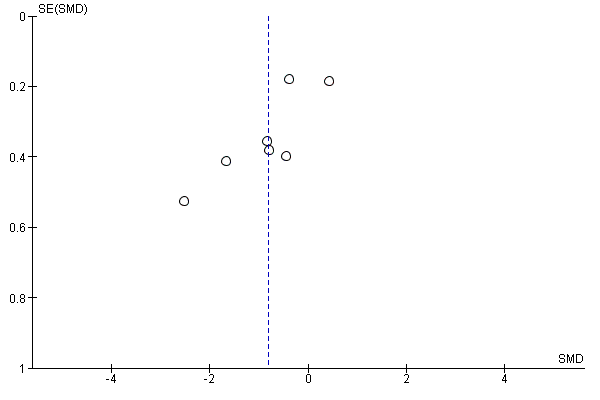
**

**G - CERT Scores for each study**


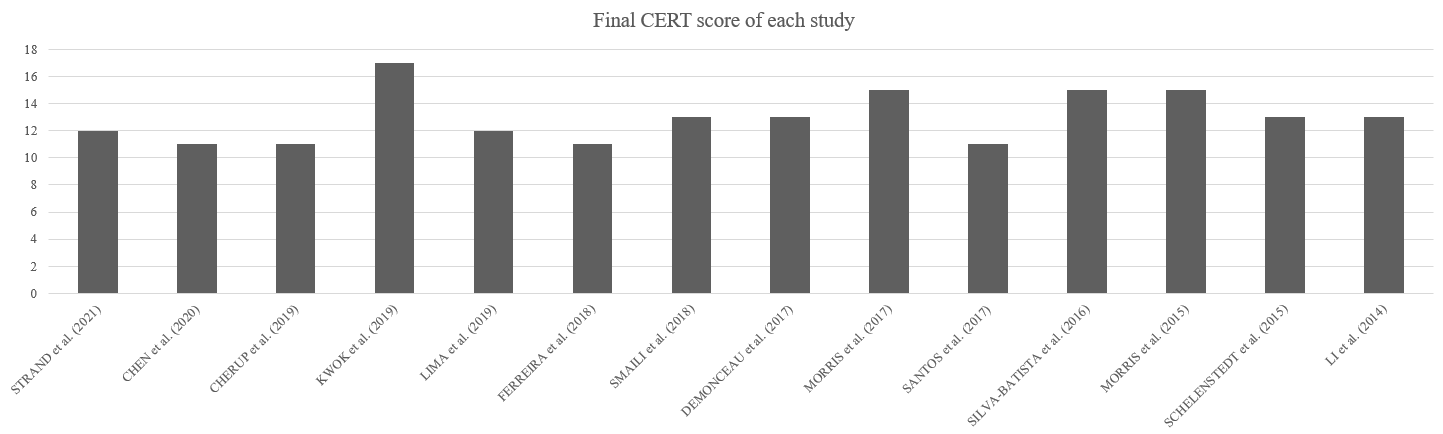


1. Horvath, K., Aschermann, Z., Kovacs, M., Makkos, A., Harmat, M., Janszky, J., ... & Kovacs, N. (2017). Changes in quality of life in Parkinson's disease: how large must they be to be relevant?. Neuroepidemiology, 48(1-2), 1-8. [↑](#footnote-ref-1)
